# Integration of force and IMU sensors for developing low-cost portable gait measurement system in lower extremities

**DOI:** 10.1038/s41598-023-37761-2

**Published:** 2023-06-30

**Authors:** Udomporn Manupibul, Ratikanlaya Tanthuwapathom, Wimonrat Jarumethitanont, Panya Kaimuk, Weerawat Limroongreungrat, Warakorn Charoensuk

**Affiliations:** 1 Department of Biomedical Engineering, Faculty of Engineering, Mahidol University, Phuttamonthon, Nakhon Pathom Thailand; 2 Faculty of Physical Therapy, Mahidol University, Phuttamonthon, Nakhon Pathom Thailand; 3 College of Sports Science and Technology, Mahidol University, Phuttamonthon, Nakhon Pathom Thailand

**Keywords:** Biomedical engineering, Physical examination

## Abstract

Gait analysis is the method to accumulate walking data. It is useful in diagnosing diseases, follow-up of symptoms, and rehabilitation post-treatment. Several techniques have been developed to assess human gait. In the laboratory, gait parameters are analyzed by using a camera capture and a force plate. However, there are several limitations, such as high operating costs, the need for a laboratory and a specialist to operate the system, and long preparation time. This paper presents the development of a low-cost portable gait measurement system by using the integration of flexible force sensors and IMU sensors in outdoor applications for early detection of abnormal gait in daily living. The developed device is designed to measure ground reaction force, acceleration, angular velocity, and joint angles of the lower extremities. The commercialized device, including the motion capture system (Motive-OptiTrack) and force platform (MatScan), is used as the reference system to validate the performance of the developed system. The results of the system show that it has high accuracy in measuring gait parameters such as ground reaction force and joint angles in lower limbs. The developed device has a strong correlation coefficient compared with the commercialized system. The percent error of the motion sensor is below 8%, and the force sensor is lower than 3%. The low-cost portable device with a user interface was successfully developed to measure gait parameters for non-laboratory applications to support healthcare applications.

## Introduction

Gait analysis is the method to qualify human walking. It aims to determine the characteristics of gait and the gait parameters. Gait analysis can be used in various fields such as sports science^[[1, 2]]^, safety^[[3–5]]^, and medicine^[[6–8]]^. Clinical gait analysis helps to identify abnormalities in walking in the elderly and to follow up on the symptoms while rehabilitating neurological disorder patients such as stroke, cerebral palsy, and spinal cord injury^[[9]]^. Gait analysis utilizes to improve the ability to walk in patients affected by sclerosis, Parkinson’s disease, and cerebellar ataxia^[[10]]^. Monitoring gait changes is useful for the early detection of some diseases and allows better treatment^[[11]]^.

Various technologies have been developed for gait analysis. The technical devices can be divided into two groups: 1. non-wearable system 2. wearable system. The non-wearable system consists of a device based on image processing, such as an infrared camera, a laser range scanner, or a time-of-flight camera to detect the motion with or without markers, and a device based on a ground sensor called a force platform to measure the ground reaction force (GRF). The wearable system uses many kinds of sensors, such as accelerometer, gyroscope, magnetometer, force sensor, flexible goniometer, and electromyography, to detect the signal of human motion^[[11]]^.

The standard gait analysis method involves combining a camera's motion capture system with a force platform^[[12, 13]]^. Nonetheless, the gait analysis system and devices, including 3D motion capture and force platform have numerous limitations, for example, the complex handling of the instrument, the error of marker placement, and the high operating cost (more than 200 K)^[[14, 15]]^, which negatively affects the detection process making it cumbersome and delayed. Sometimes it requires specialists who have a high level of technical training skills to operate the system. The limitations of the force platform are that it cannot directly measure the applied force vector and requires many force platforms to measure the numerous steps of walking^[[11]]^. Another limitation is that the system is restricted to the laboratory, so it cannot capture the gait pattern in daily activities. Therefore, many researchers try to develop the lower cost wearable gait measurement device to support healthcare applications in daily activities^[[16, 17]]^.

Although motion capture is used to be the gold standard for gait analysis, many researchers prefer wearable sensors such as inertial measurement unit sensors (IMU)^[[18–21]]^. IMU sensor consists of a combination of accelerometer, gyroscope, and magnetometer. IMU sensors can analyze gait, joint angle kinematics, and body motion. The IMU-based system has many advantages, for example, lower cost, portable, user friendly, suitable for daily activities, and easy to calibrate compared with motion capture system^[[19, 22]]^. Md. Mahmudur Rahman et al. presented the utilization of IMU sensor to measure the joint angle and the motion data during the exercise to improve the treatment outcomes^[[23]]^. Ming Gui Tan et al.^[[24]]^ presented the development of a low-cost sensor system by using an accelerometer, force-sensitive resistors, and EMG electrodes to measure gait parameters. Abu Ilius Faisal et al.^[[25]]^ presented a low-cost gait analyzer by using inertial sensors to detect gait characteristics. Some researchers evaluate gait by using inertial sensors: accelerometer, gyroscope, and magnetometer^[[15, 26, 27]]^.

Instrumented insoles based on force sensors, including piezoelectric sensors, capacitance sensors, piezoresistive sensors, and force-sensitive resistance (FSR) sensors, are used to measure ground reaction force in gait analysis. The most commonly used sensor in various researches is the FSR sensor^[[28]]^. Based on the study by Anas M. Tahir et al., it is reported that the FSR sensor is the most effective sensor for use in smart insoles compared to piezoelectric sensors^[[29]]^. It has flexible characteristics, does not disturb gait patterns, consumes low power, and is inexpensive^[[30]]^. It can also be used in everyday activities.

However, IMU and FSR in gait analysis have some limitations. IMU has some problems involving error contamination by the gravitational force in the accelerometer. The error of the double-integrated acceleration to determine the position. The noise signals surrounding the environment can affect the magnetometer^[[19, 22]]^. The limitations of the FSR are that it varies depending on the surface of insoles or shoes, and the position misalignment on the insole may not match the point of applied force due to the small size of the FSR^[[18, 31]]^.

This study presents the integration of adapted force sensors and IMU sensors for developing a low-cost portable gait measurement device and system suitable for outdoor activities. The development of the device and system is considered to decrease the previous limitations of IMU and FSR sensors. In IMU sensors, accelerometer, gyroscope, and magnetometer are integrated into a single device to reduce measurement error and increase the precision of the measuring movement^[[23]]^. In addition, the Kalman filter method, which is the ideal filtering method with minimum mean squared error and remains unchallenged in industrial applications^[[32–34]]^, has been used to eliminate the noise from the movement. In GRF detection, this research focuses on the flexible force sensor, which has many models suitable to be specially shaped and adapted for integration into the insole to avoid position misalignment. The sensor is used to estimate GRF in outdoor activities and can measure GRF in several walking steps simultaneously with detecting joint angles in dynamic analysis. The developed system can measure acceleration, angular velocity, magnetic fields, and ground reaction force. All data are used to calculate the gait parameters such as ground reaction force, hip joint angle, knee joint angle, and ankle joint angle and validated by commercialized devices: Motive-OptiTrack camera (NaturalPoint, Inc., United States) and MatScan (Tekscan, Inc., United States). This research aims to develop an accurate measurement device to support healthcare applications that benefit from the early detection of abnormal gait in daily activities. The developed devices and systems that are low-cost, portable, high-accuracy, reliable, and non-invasive are preferred.

## Methods

The developed device and system are designed to measure gait parameters such as ground reaction force, acceleration, angular velocity, and angle of joints. The system consists of seven IMU sensors and fourteen flexible force sensors (seven sensors in each insole). Five IMU sensors are designed in five small rectangular boxes which can be easily on the rubber band. Another two IMU sensors combined with force sensors in the insole are designed in small boxes located on shoes. To develop the device, the processes are divided into three parts: 1. Motion detection 2. Plantar force measurement, and 3. User interface.

### Motion detection

GY-85 IMU sensor (Shenzhen Gintech Electronic Trade Co., Limited, China) is a 9-degree freedom sensor fusion, which consists of an accelerometer (ADXL345), gyroscope (ITG3200), and magnetometer (HMC5883L) integrated into the integrated circuit. The sensor has 17 mm × 22 mm × 2 mm in size. The operating temperature is between − 40 °C and 85 °C. The sensor uses the I2C communication protocol, and is supplied with 3–5 voltages. GY-85 connects with Lolin32 Lite Wemos (Espressif Systems, China) microcontroller to process the data. Lolin32 Lite Wemos based on ESP32 (WROOM32: Xtensa dual-core 32-bit LX6 microprocessor) integrates with antenna, power amplifier, filter, 2.4 GHz dual-mode WIFI, and Bluetooth chips functionality through its SPI/SDIO or I2C/UART to interface with other systems. The size of Lolin32 Lite is 50 mm × 25.4 mm × 7 mm. The module provides 3.3 V for operation. The microcontroller and IMU sensor are integrated into a circuit that is easy to use. A lithium battery of 3.7 V is used to supply the circuit board.

Acceleration, angular velocity, and magnetic field from the IMU sensor are used to calculate roll (the rotation around X axis angle), pitch (the rotation around Y axis angle), and yaw angle (the rotation around Z axis angle)^[[35, 36]]^. Due to the Earth's gravity accelerometer being sensitive in the downward direction, acceleration data are used to calculate roll and pitch. The yaw angle is calculated by using Earth’s magnetic field. However, the raw data of 3-axis acceleration from the accelerometer, 3-axis angular velocity from the gyroscope, and 3-axis magnetic field from the magnetometer are retained to analyze other parameters.

The algorithm to calculate the hip, knee, and ankle angle is programmed in the microcontroller. The Arduino program is used for programming the code to the ESP board. The processes start with power on the chips. Then the sensor activates the movement detection in 3 axes (XYZ). The sensor is calibrated to perform offset and gain calculations before seeing updated results. The sensors' data are filtered using lowpass, and Kalman filters to eliminate the error. The conversion of acceleration to g unit uses Eq. (1). Acceleration data from the sensor always has some noise, so a low-pass filter implemented in Eq. (2) is used to reduce it. Kalman filters are also used to smooth the data from the IMU sensor for obtaining accurate orientation. The Kalman filter library in Arduino is used.

1 $${G}_{Accel}= {Raw}_{Accel}\times \frac{Range}{{2}^{Resolution}-1}$$

2 $${y}_{t}=\left(\alpha \times {x}_{t}\right)+\left(1-\alpha \right)({y}_{t-1})$$

where y_t_ is the low pass filtered signal, y_t-1_ is the previous filtered signal, x_t_ is the accelerometer reading, and the α smoothing factor

**Figure 1:**
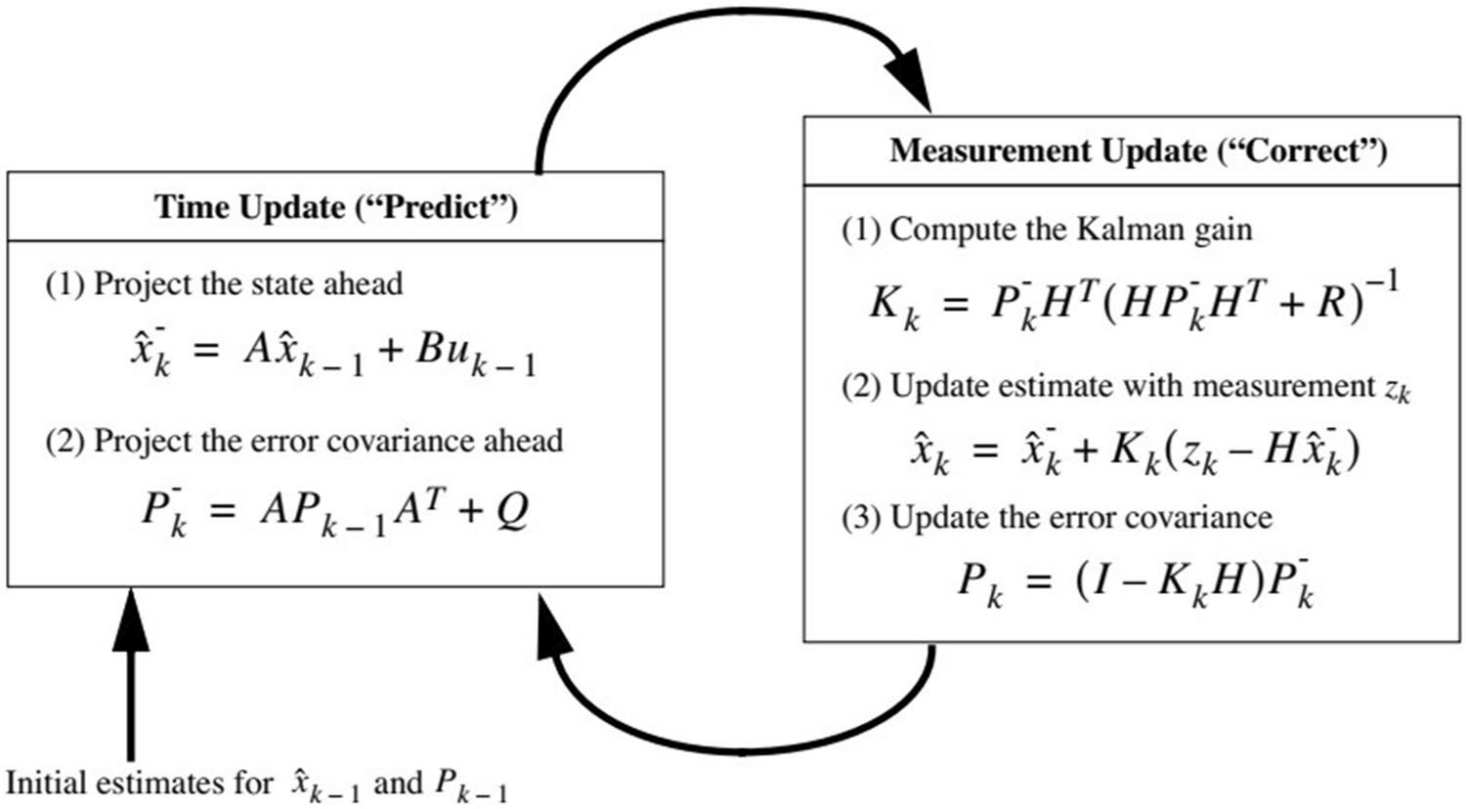
The Kalman filter operation.

The Kalman filter method^[[37]]^ is the cycle of the predicted next state of the dynamic system (time update) and is corrected with the observation model (measurement update). The current state (x^−^_k_) and error covariance estimates (P^−^_k_) are projected forward (in time step k − 1 to step k) by the time update equations to produce the a priori estimates for the following time step. A is the state transition matrix. B is a control matrix. u is the input variable. Q is the process noise covariance. R is the measurement covariance. The measurement update equations handle the feedback (adding a new measurement to the a priori estimate to get better posteriori estimate x_k_). The first task of measurement update is to compute Kalman gain (K_k_) and then update the estimate with actual measurement z_k._ to obtain a posteriori state estimate_._ The last step is to get the posterior error covariance estimate (P_k_). The steps of using the Kalman filter are presented in Fig. 1^[[37]]^.

The filtered acceleration data are calculated to roll and pitch angle by using Eq. (3).

3 $$Roll = \arctan \frac{{ - G_{x} }}{{G_{z} }},Pitch = arctan\frac{{G_{y} }}{{\sqrt {G_{x}^{2} + G_{z}^{2} } }}$$

where G_x_ is acceleration data (g unit) in the X axis, G_y_ is acceleration data (g unit) in the Y axis, G_z_ is acceleration data (g unit) in the Z axis.

The angular velocity in XYZ axes is calculated from reading data from the gyroscope by Eq. (4).

4 $${angle}_{t}= {angle}_{t-1}+ (angula{r}_{rate}\times looptime)$$

where angle_t_ is the angle in degree unit and angle_t−1_ is the previous angle in degree unit

The yaw angle is calculated by magnetic field data (micro-Tesla unit) and then the declination angle is added, which is the error of the magnetic field in the stayed location, following Eq. (5). The declination angle in any location can be found at http://www.magnetic-declination.com/.

5 $$Yaw=\mathrm{arctan}\frac{-{Y}_{h}}{{X}_{h}}+declination$$

where X_h_ is the horizontal earth magnetic field in the X axis, Y_h_ is the horizontal earth magnetic field in the Y axis.

The roll, pitch, and yaw angle provide the following ranges [− 180°,180°], [− 90° to 90°], and [0° to 360°], respectively. All of these angles are used to estimate hip joint, knee joint, and ankle joint angles in the experiment.

### Plantar force measurement

The flexible force sensor is the main part of measuring plantar force distribution. In this research, flexiforce sensors A401 and A502 models (Tekscan, Inc., United States) are integrated with the comfortable insole to measure force in the Newton unit. The flexiforce sensor is thin, flexible, lightweight, high accuracy, simple to use, cost-effective, and easy to customize. Flexiforce A401 has a 56.9 mm length, 31.8 mm width, and 0.2 mm thickness. It has a 25.4 mm diameter in the sensing area. Flexiforce A502 has an 81.3 mm length, 55.9 mm width, and 0.2 mm thickness. It has 50.8 mm × 50.8 mm of the sensing area. The maximum force range of these sensors is up to 44,482 N. The characteristic of this sensor is that it can change the dynamic force range by adjusting the driven voltage and the resistance of the feedback resistor. The sensor generates voltage output following the force placed on the sensing area of the sensor. Voltage output is converted to force by force calibrating equation. Each force sensor is calibrated by using a force gauge (IMADA, Inc., Japan). The force gauge is a highly accurate instrument for force measurement applications. The range of force is 1000 N with an accuracy of ± 0.2%. It has a 30,000/s ultra-high sampling rate and runs on internal Ni-MH batteries or AC adapters.

**Figure 2:**
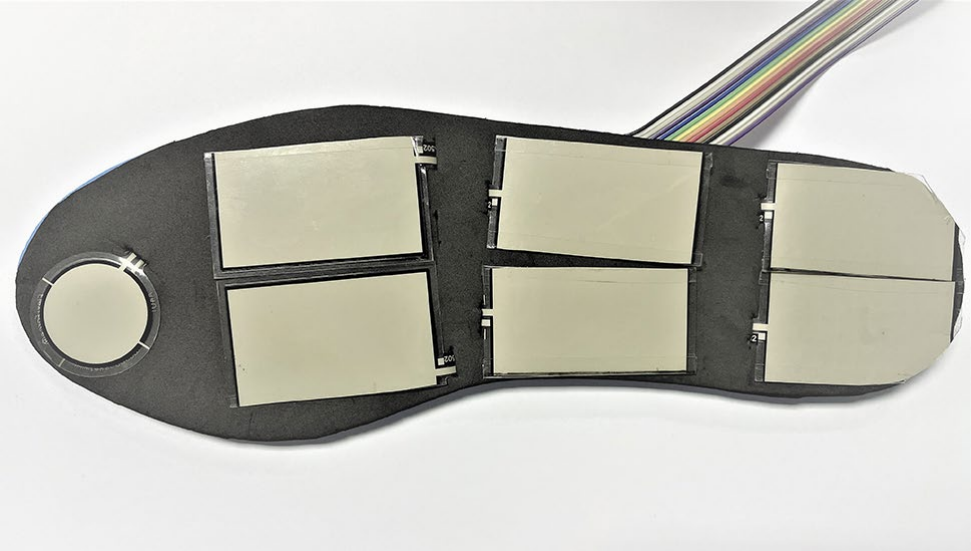
Customized force sensor with insole.

To customize force sensors (Fig. 2), the sensors are cut to the size and shape that can fit the insole. Seven force sensors are placed on each insole in seven areas that usually have high pressure while walking which are the toe, medial and lateral metatarsal, medial and lateral midfoot, and medial and lateral heel. Force sensors connect with microcontroller ESP32-S2-DevKitM-1 (Espressif Systems, China) for processing the data. ESP32-S2-DevKitM-1 is the development board with a WIFI development tool. It has I2C, I2S, SPI, and UART interface types. The size of the module is 54 mm × 25.4 mm × 7 mm. The module provides 3.3–5 voltages for operation. The data from all sensors are processed in microcontrollers and transmitted via WIFI to display and store in the user interface on a laptop.

### User interface

User interface is developed by using the Visual Studio C# program to receive and display the data from motion sensors and force sensors. WIFI is the communication protocol in this project. The user interface is designed to have 7 ports to receive the data from each sensor. The user interface is designed to show the absolute angle from the IMU sensor in three axes and the force from force sensors in each insole. The user interface is designed to have a save button to save the data from the sensors. The data that are stored are three-axis acceleration, three-axis angular velocity, three-axis magnetic field, roll angle, pitch angle, yaw angle, and force from seven areas of each insole.

## Experiments

**Figure 3:**
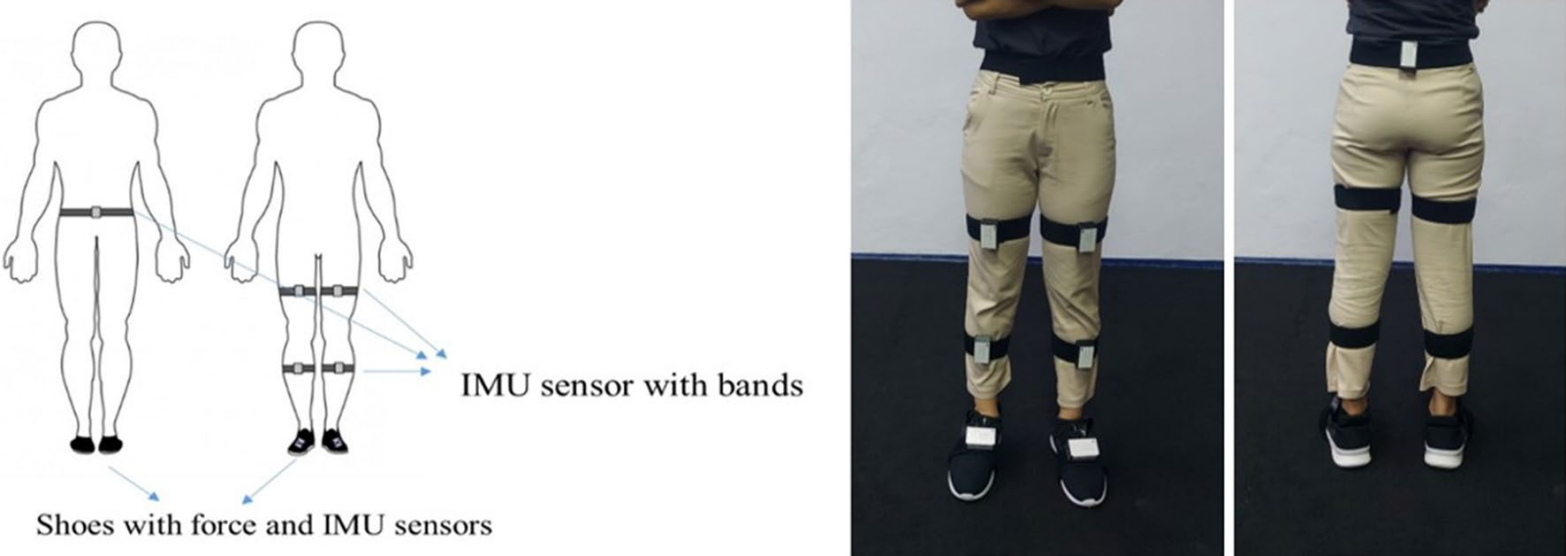
The locations of the motion sensors and shoes.

In gait analysis, the system is designed to have seven motion sensors which have the IMU sensor and circuit in each box. Five motion sensors are designed to locate at the lumbar, right and left thighs, and right and left shanks. Another two motion sensors connect with the integrated insole with force sensors located at the right and left insteps of shoes. The locations of the motion sensors and shoes are shown in Fig. 3. The experiments are separated into three sections: 1. Performance testing of the force sensors 2. Performance testing of motion sensors 3. Dynamic testing of the motion sensors and force sensors.

### Performance testing of the force sensors

A force gauge was used to test the performance of force sensors. Different ranges of force are used to test the accuracy and precision of the force sensor. Each force level is repeatedly tested 15 times. The data in the Newton unit are saved in the user interface. The data are analyzed to find the min, max, mean, SD, and percent error of the sensor as shown in Table 1. The result shows that the accuracy of force measurement is better than 97%.

**Table 1:** Descriptive statistic of force sensor.

Force (N)	Reading data (15 times)		Mean	SD	%Error
	Min (N)	Max (N)			
50	45	55	50.43	2.31	0.86
100	98	109	102.57	3.06	2.57
150	148	160	151.93	3.67	1.29
200	195	207	202.14	3.32	1.07
250	247	259	251.43	3.11	0.57
300	295	315	301.57	4.97	0.52
350	348	360	352.64	3.32	0.76
400	390	411	401.43	5.58	0.36
450	446	463	451.36	4.03	0.30
500	494	510	500.50	3.88	0.10
600	597	607	600.50	3.06	0.08
700	700	714	704.86	4.02	0.69
800	791	809	802.07	4.76	0.26

### Performance testing of motion sensors

Goniometer and Motive-OptiTrack camera system (NaturalPoint, Inc., United States) are used to test the performance of IMU sensors to measure motion angle repletely ten times in each angle. The Motive-OptiTrack camera system consists of eight infrared cameras model S250e and OptiTrack software. The resolution is 832 × 832 (0.7 MP). The frame rate is 30–250 FPS. The latency is 4 ms. Ethernet syncs all cameras. Motion sensors and markers are placed on the goniometer and set to different angles which include 30°, 60°, 90°, 120°, 150°, and 180° to measure and compare roll, pitch, and yaw angles as shown in Table 2a–c respectively. The result shows that the percent error is less than 8%.

**Table 2:** Descriptive statistic of (a) roll angle of IMU sensor, (b) pitch angle of IMU sensor, (c) yaw angle of IMU sensor.

Goniometer angle	Motive-OptiTrack camera				IMU sensor				%Error	
	Min	Max	Mean	SD	Min	Max	Mean	SD	IMU compared with goniometer	IMU compared with motive-OptiTrack
(a) Roll angle										
30	30.10	30.61	30.32	0.17	31.14	32.22	31.73	0.41	5.75	4.63
60	60.11	60.72	60.43	0.16	61.06	62.15	61.67	0.40	2.79	2.06
90	90.23	90.46	90.35	0.07	88.32	88.81	88.61	0.17	1.55	1.93
120	120.44	120.76	120.64	0.10	117.55	118.44	117.82	0.27	1.82	2.34
150	149.52	150.28	149.95	0.23	147.19	149.52	148.18	0.83	1.22	1.18
180	177.60	178.86	178.44	0.37	178.86	180.38	179.65	0.60	0.20	0.68
(b) Pitch angle										
30	30.28	30.63	30.50	0.12	32.35	32.68	32.48	0.12	8.27	6.49
60	61.11	61.55	61.33	0.11	61.67	63.08	62.33	0.49	3.89	1.63
90	90.89	91.72	91.19	0.26	90.26	91.42	91.02	0.35	1.13	0.20
120	120.73	121.13	120.96	0.13	123.05	124.95	123.82	0.53	3.18	2.36
150	149.64	150.52	150.25	0.25	144.95	154.13	149.13	3.51	0.58	0.74
180	178.33	179.03	178.80	0.20	172.76	174.95	173.28	0.64	3.74	3.09
(c) Yaw angle										
30	29.77	30.73	30.09	0.27	31.99	33.02	32.30	0.34	7.65	7.34
60	60.22	60.65	60.41	0.16	62.07	62.92	62.49	0.30	4.15	3.45
90	89.88	90.31	90.10	0.13	93.26	93.49	93.41	0.07	3.79	3.67
120	120.10	120.57	120.36	0.14	115.72	116.89	116.40	0.33	3.00	3.29
150	149.97	150.45	150.18	0.16	148.00	148.42	148.13	0.13	1.25	1.37
180	178.03	178.33	178.22	0.09	179.96	180.83	180.27	0.27	0.15	1.15

### Dynamic testing of the motion sensors and force sensors in walking activity

The experiment is set up to compare the result of joint angle measurement between the IMU sensors and Motive-OptiTrack system and to compare the results of ground reaction force between the insoles system and MatScan (Tekscan, Inc., United States) in walking activity. For Motive-OptiTrack system, it consists of eight cameras that are calibrated and set up in the gait laboratory. The system is commercialized which can be represented as the standard in this experiment. MatScan is the foot pressure measurement platform that captures static and dynamic pressure measurement data. It consists of 2288 sensels and can measure pressure up to 862 kPa. The size of the sensors is 435.9 mm × 368.8 mm. The scanning speed is up to 100 Hz. The systems are commercialized and can be used as reference systems in this experiment. The informed consent was obtained from all subjects. The experiment procedure can be described in 5 steps.

**Figure 4:**
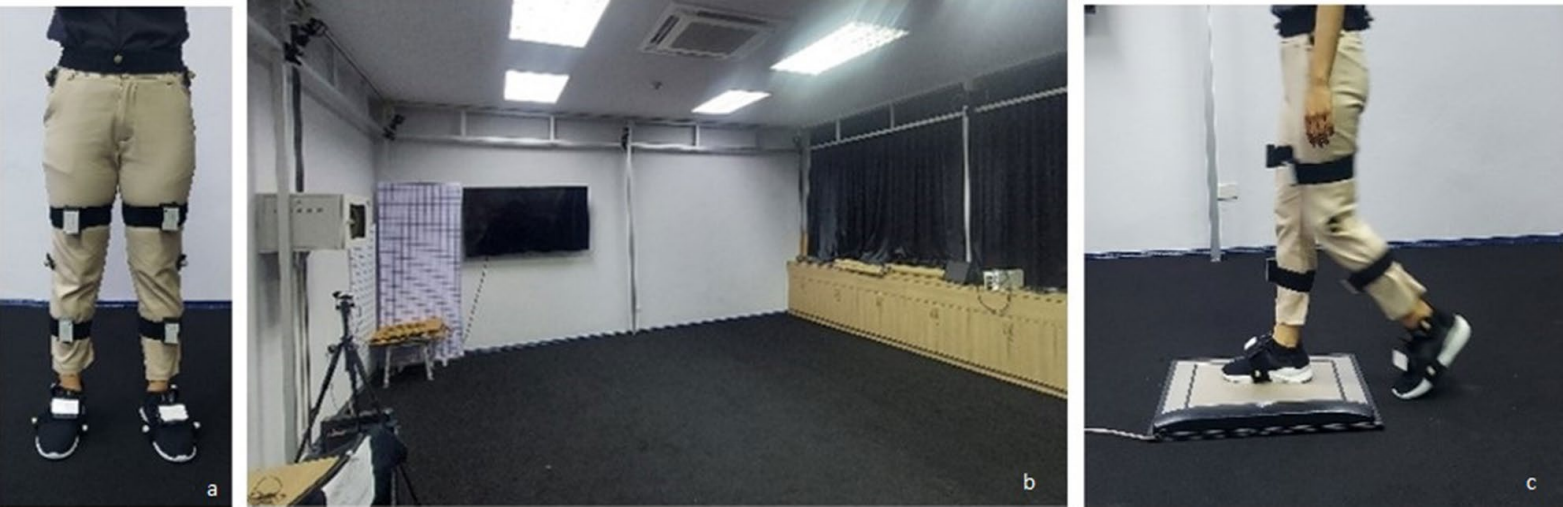
(**a**) Wearable sensors and shoes with (**b**) Motive-OptiTrack and (**c**) MatScan systems.

1. The normal subject was asked to wear wearable sensors and shoes and to put markers on the lower body. The physical therapist is requested to put the markers on the subject body to ensure that the markers are in the right place.
2. The Motive-OptiTrack and MatScan system is calibrated and set to be ready to perform the experiment (Fig. 4).
3. Before testing, IMU sensors and force sensors are reset to eliminate the zero drift of the sensors. Two systems are simultaneously recorded. The orientation of the developed system and the reference systems are set to correspond to each other.
4. The experiment starts with the subject standing still for 5 s, walking straight for three cycles (six steps), then standing still for another 5 s. The subject walk passes MatScan in the third step.
5. The subject is asked to perform the experiment five times.

The data for each step of walking are analyzed later. The results of the two systems are analyzed by SPSS to find the correlation coefficient of knee angle, hip angle, and ankle angle in the XYZ axis and the correlation coefficient of ground reaction force.

### Ethics approval and consent to participate

The study protocol was approved by the Mahidol University Central Institutional Review Board (reference number: MU-CIRB: 2021/294.0406), and the study was conducted in accordance with the Declaration of Helsinki.

## Results

**Figure 5:**
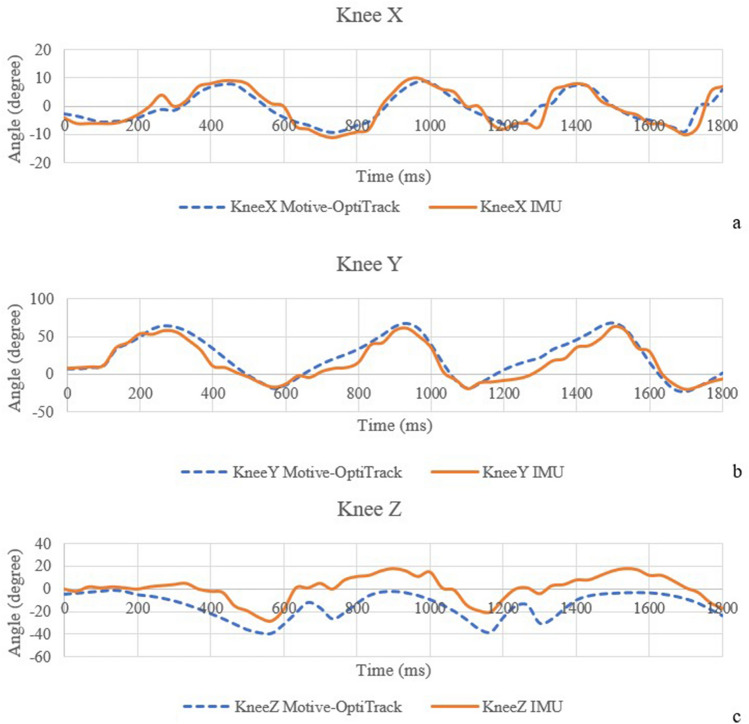
The comparison of knee angle between the IMU sensor and Motive-OptiTrack, (**a**) X-axis (**b**) Y-axis (**c**) Z-axis.

**Figure 6:**
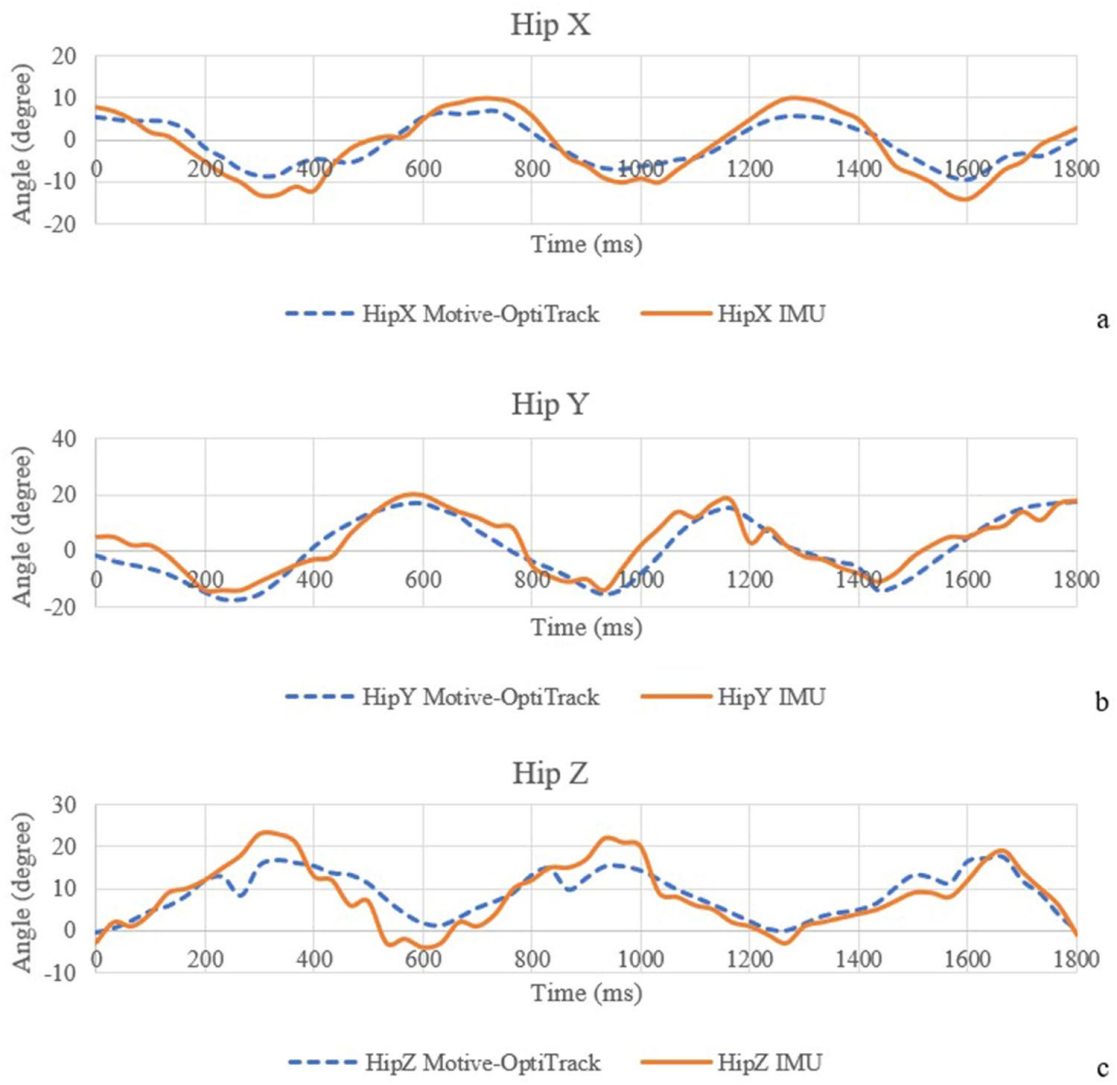
The comparison of the hip angle between the IMU sensor and Motive-OptiTrack, (**a**) X-axis (**b**) Y-axis (**c**) Z-axis.

**Figure 7:**
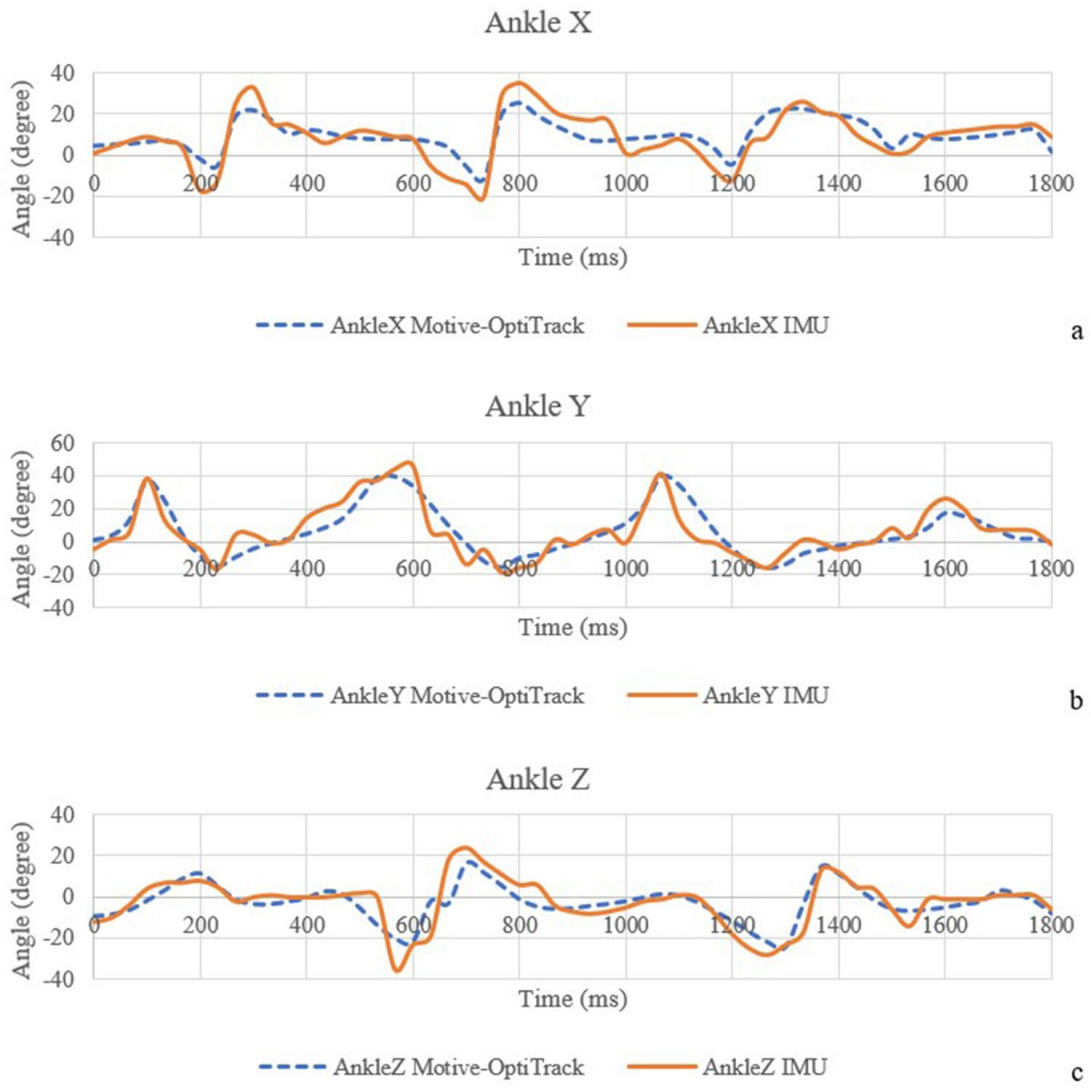
The comparison of ankle angle between the IMU sensor and Motive-OptiTrack, (**a**) X-axis (**b**) Y-axis (**c**) Z-axis.

**Figure 8:**
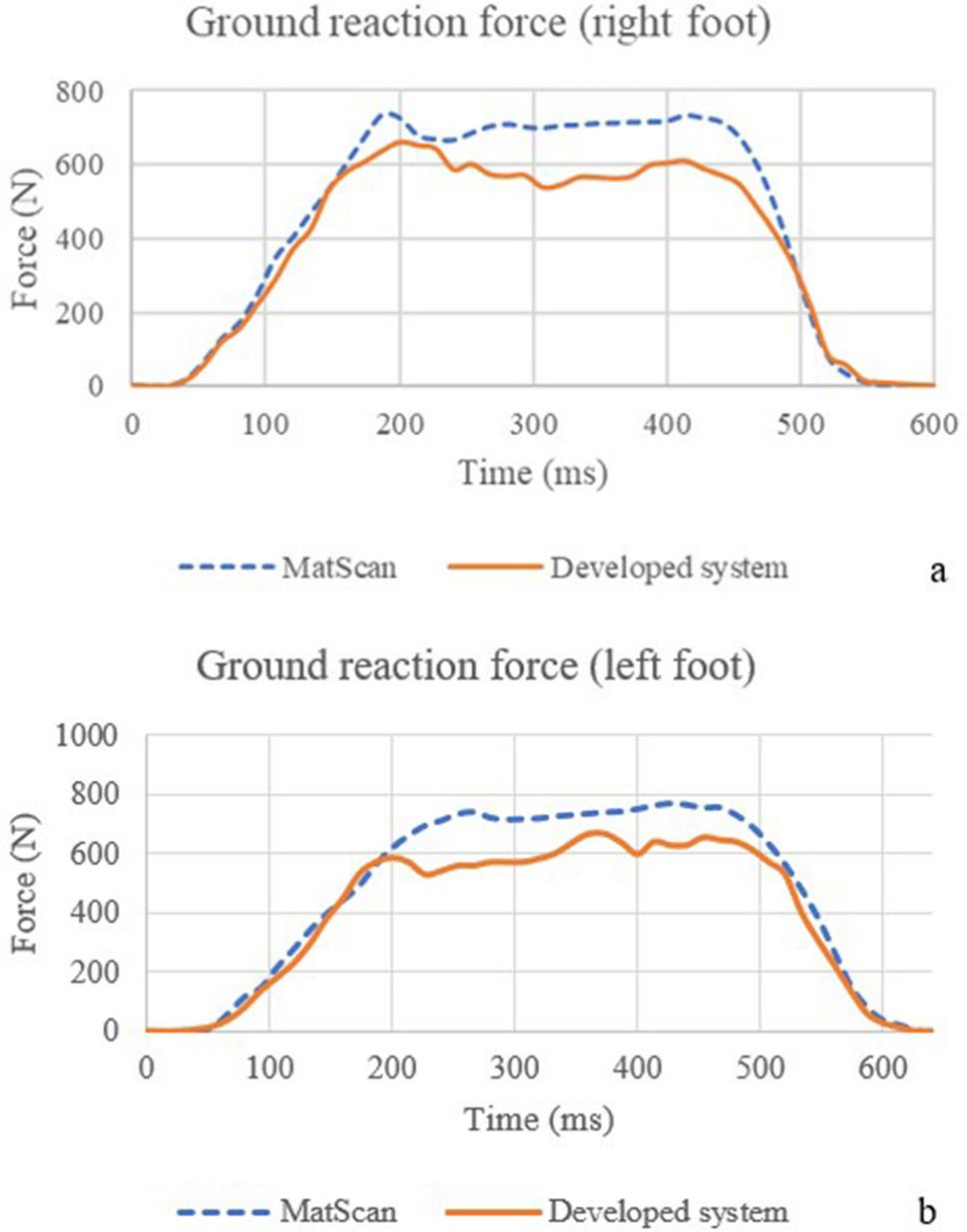
The comparison of the ground reaction force of (**a**) right foot and (**b**) left foot between the developed system and MatScan.

The correlation coefficients of the IMU sensor—Motive-OptiTrack and the correlation coefficients of the developed system—MatScan are presented in Table 3. Figure 5 shows the comparison of knee angle between the IMU sensor and Motive-OptiTrack, (a) X-axis (b) Y-axis, and (c) Z-axis. The X-axis of the knee angle represents knee adduction and abduction angles. The Y-axis of the knee angle represents knee flexion and extension angles. The Z-axis of the knee angle represents the knee internal rotation and external rotation angles. Figure 6 shows the comparison of the hip angle between the IMU sensor and Motive-OptiTrack, (a) X-axis (b) Y-axis, and (c) Z-axis. The X-axis of the hip angle represents hip adduction and abduction angles. The Y-axis of the hip angle represents hip flexion and extension angles. The Z-axis of the hip angle represents the hip rotation angle. Figure 7 shows the comparison of ankle angle between the IMU sensor and Motive-OptiTrack, (a) X-axis (b) Y-axis (c) Z-axis. The X-axis of the ankle angle represents ankle inversion and eversion angles. The Y-axis of the ankle angle represents dorsiflexion and plantarflexion angles. The Z-axis of the ankle angle represents ankle adduction and abduction angles. Figure 8 shows the comparison of the ground reaction force of (a) the right foot and (b) the left foot between the developed system and MatScan.

**Table 3:** The correlation coefficients of IMU sensor—Motive-OptiTrack and the correlation coefficients of the developed system—MatScan.

Knee X	Knee Y	Knee Z	Hip X	Hip Y	Hip Z	Ankle X	Ankle Y	Ankle Z	GRF right	GRF left
Correlation coefficient										
0.94	0.96	0.82	0.95	0.92	0.87	0.87	0.87	0.84	0.99	0.99

Following the interpretation of correlation coefficients of Patrick Schober et al., research^[[38]]^, the level of the correlation coefficient can be interpreted as 0.00–0.10 is negligible correlation, 0.10–0.39 is weak correlation, 0.40–0.69 is moderate correlation, 0.70–0.89 is strong correlation, and 0.9–1.00 is very strong correlation.

The results of knee X, knee Y, hip X, and hip Y can be interpreted to possess a very strong correlation. The results of knee Z, hip Z, ankle X, ankle Y, and ankle Z can be interpreted as having a strong correlation. The result of the ground reaction force of the right foot and left foot can be interpreted as a very strong correlation.

## Discussion

IMU-based angle and position computation are usually disturbed by noise. The position and angle errors are known as drift, the term for the steadily increasing departure from the actual position. There are several techniques used to minimize the drift. The ZUPT algorithm, also known as the zero-velocity update algorithm, is a considerably more straightforward technique. The approach applies a correction to the location computations while a gait is stationary (zero velocity). However, due to the undetected location error and azimuth misalignment angle, ZUPT could be more effective. The azimuth misalignment angle of ZUPT can be estimated using the magnetometer as an external sensor. The magnetic field strength measurement could be used to estimate the foot heading if the magnetometer were additionally positioned on the foot. However, due to the measurement error brought on by the magnetic dispersion, the pedestrian cannot use this measurement as a guide to calculate the azimuth misalignment angle^[[39]]^. The undetected position error and azimuth misalignment angle cause an ongoing increase in azimuth error and a continual increase in positioning error, while ZUPT can rectify the velocity error and horizontal misalignment angle. This means that while the position error brought on by azimuth error can be corrected, the position error brought on by velocity error and horizontal misalignment angle cannot. A method for introducing inequality limits into the Kalman filter is suggested by Isaac Skog^[[40, 41]]^. Lowpass filter and Kalman filter are used to eliminate the noise in the IMU sensors. The result of the motion sensor shows that the developed device can achieve higher than 92% accuracy in static measurement. In dynamic measurement, the result shows a strong correlation coefficient (0.7–0.89) in knee Z, hip Z, ankle X, ankle Y, and ankle Z compared with commercial devices. The correlation coefficients of knee X, knee Y, hip X, and hip Y are more than 0.9, which can be interpreted as a very strong correlation. However, there are some concerns while placing the IMU sensor on the lower part of the body. The motion sensor needs to be tightly attached to the body parts because the shaking of the IMU sensor can cause errors in data.

Measuring GRF is helpful in many applications, such as classifying the type of walking to diagnose the presence of disorder^[[42]]^, detecting the abnormal pressure distribution^[[43]]^, and calculating the center of pressure (COP)^[[44]]^. The change in GRF force provides knowledge concerning post-stroke gait^[[45]]^ and knee osteoarthritis^[[46]]^. This study shows that the developed device has high accuracy (> 97%), which can be used to measure ground reaction force. However, in Fig. 8, there are differences between the two values of ground reaction force measured by MatScan and developed shoes. The GRF value measured by MatScan seems to have a higher force value than that measured by developed shoes. This may be because some areas of the insole have no force sensors, such as second to fifth metatarsal heads, and some gaps exist between the force sensors. The result may be erroneous if the plantar force is applied in these areas. Some research also has similar problems. Instrumented insoles with few sensors frequently have poor mediolateral axis accuracy^[[47]]^. Hence, the location of force sensors should be designed to be distributed to cover whole areas of the insole to minimize errors. Another possible cause is that the shoes' size may not match the subject foot. Hence, the insole with force sensors should be designed to have many sizes to match the subject foot.

## Conclusion

In this research, the low-cost portable motion and force measurement system was successfully developed with high accuracy and high performance to estimate the gait parameters, such as ground reaction force and lower limb joint angles, for daily activities. The user interface is easy to use and practical to record and store the data. The wireless protocol is stable for data transfer from the device to the laptop. The developed system is useful in both indoor and outdoor applications. The cost of the system is much lower than the commercial products. Future work will involve this device to measure gait parameters and compare the difference between normal and overweight subjects.

## Data Availability

The data that support this study are available from the corresponding author upon reasonable request.
